# Radiological features and treatment of erupting maxillary canines in relation to the occurrence of dental developmental abnormalities

**DOI:** 10.2340/aos.v83.40488

**Published:** 2024-04-25

**Authors:** Jenni Ristaniemi, Terhi Karjalainen, Kati Kujasalo, Wille Rajala, Paula Pesonen, Raija Lähdesmäki

**Affiliations:** a Oral Development and Orthodontics, Research Unit of Population Health, Faculty of Medicine, University of Oulu, Oulu, Finland; b Infrastructure for Population Studies, Faculty of Medicine, University of Oulu, Oulu, Finland; c Oral and Maxillofacial Department, Medical Research Center Oulu (MRC Oulu), Oulu University Hospital, Oulu, Finland

**Keywords:** Abnormal eruption, developing dentition, diagnostics, human, mixed dentition

## Abstract

**Objective:**

This study aimed to investigate the radiological features of the permanent canines and the treatment carried out to ensure their eruption relative to certain features involved in Dental Anomaly Patterns (DAP).

**Material and Methods:**

The cross-sectional part of this retrospective register-based study focused on 1,315 dental panoramic tomographs (DPTs) of children aged 8.5–10.5 years, while the longitudinal part involved information on the treatment provided for 1,269 canines after the DPTs and on their eruption into the oral cavity.

**Results:**

The canines of the DAP children more often showed evidence of early treatment (*p* = 0.014), with girls having more frequently interceptive treatment (*p* = 0.004) and boys early headgear (*p* = 0.022). Delayed dental age was associated with early treatment (OR 3.29, 95% CI 1.08–9.99). Either no or clear overlapping of a canine with the lateral incisor occurred more often in the DAP children, whereas canine inclination did not differ between the groups. The root development stage of the canine was more often either beginning or well advanced in the DAP children.

**Conclusions:**

The children with dental developmental abnormalities more often showed evidence of early treatment for the canines. Monitoring of the erupting canines after the first mixed stage is important to enable timely early treatment.

## Introduction

The development of the human dentition involves a number of abnormalities leading to clinical problems. One of the most problematic conditions is eruption disturbance in a maxillary permanent canine, since the eruption route is long and complex [1,2] and the eruption process shows many kinds of abnormalities [3]. Normally the maxillary canines erupt into the oral cavity at an age of 10–12 years, during the late stage of the mixed dentition, but with a normal deviation of several years [4].

Overlapping with the lateral incisor root and a larger mesial inclination angle of the maxillary canine are elements in the earlier stages of the eruption and development of the canines and both should be reduced if eruption is to proceed normally [5–7], whereas in an abnormal eruption path both will still be pronounced [6, 8, 9]. Root development does not differ between normally and abnormally erupting maxillary canines [6, 9].

Early diagnosis of eruption disturbances in the maxillary canines in relation to the stage in dental development is crucial so that early interceptive treatment can be performed effectively and in time. Extraction of a primary maxillary canine [10–13] and the use of headgear in the early mixed dentition [14, 15] are effective treatment options for managing eruption disturbances in the permanent canines.

Certain dental developmental abnormalities have been reported to occur together more often than can be explained by chance alone [16–22]. It was Peck who first described the concept of Dental Anomaly Patterns (DAP) [17, 23–30]. Many of these conditions can be diagnosed during the early stages of the mixed dentition, providing an opportunity to monitor features of DAP that may possibly occur later. Eruption disturbances in the maxillary canines have been linked to DAP, as palatally displaced maxillary canines, for example, have been stated to be one DAP feature [17, 24].

Dental panoramic tomograph (DPT) is a basic examination for assessing the developing dentition and observing the eruption of the permanent maxillary canines. The aim of the present study was to investigate the radiological features of the erupting canines a couple of years before eruption into the oral cavity and the treatment provided for the canines in relation to the dental developmental abnormalities involved in DAP as seen in a DPT. Our hypotheses were that children with the dental developmental abnormalities studied here have pronounced overlapping of the maxillary canines with the lateral incisors and larger inclination angles in the maxillary canines, and that such children more often receive treatment for a maxillary canine.

## Materials and methods

### Study material

The cross-sectional part of this register-based research included 1,454 digitally copied DPTs of the developing permanent dentition from a cohort of third-year primary school children of Finnish ancestry in Eastern Finland born between 1980 and 1996. For the longitudinal part of this research, the dental records and other dental radiographs were examined in order to determine the treatment carried out for the maxillary canines. The data were gathered retrospectively from the Health Centre’s dental records with permission (2006, 2015 and 2019) from the keeper of the register. All personal information was coded to prevent identification.

### DAP features

The features listed below were introduced first as DAP by Peck [17] and were looked for in the DPTs studied here. Their prevalences have been reported earlier [22].

#### Absent teeth

A permanent tooth was deemed congenitally absent if the follicle was not visible. Absent teeth were assessed by ER and in cases of rare absences (maxillary canines, mandibular incisors and second molars) confirmed by JR. Third molar absences were excluded.

#### Microform teeth (peg-shaped maxillary lateral incisors)

Peg-shaped lateral incisors were recorded when the mesiodistal width of the crown was smaller than that of the cervical part of the tooth [31], the anatomical shape of the crown was pointed in form and the root was possibly smaller [32]. Peg-shaped maxillary lateral incisors were assessed by JR, and a consensus opinion was sought by JR and RL when necessary. The one exclusion criterion was an unclear DPT. Calibration of the DPT material was performed by JR and borderline cases (*n* = 14) discussed with RL.

#### Delay in tooth formation and eruption (generalized)

Dental age was assessed in terms of the delay (≤ -1 year) relative to the chronological age. Dental age was determined by EM and JI using the seven teeth dental maturity method of Demirjian [33, 34] and assessed by reference to Finnish maturity curves [35]. The exclusion criteria were an absent tooth (no counterpart) or poor quality of the DPT. The two examiners assessed the same set of 30 DPTs twice to test the accuracy of the assessments.

#### Infraocclusion (of primary molars)

The primary molars were assessed relative to the occlusal plane and the marginal ridges of the adjacent teeth, and the DPTs were categorized as showing no visible infraocclusion or visible infraocclusion in at least one primary molar in the maxilla or mandible, assessed by JR and calibrated by JR and RL. The exclusion criteria were an absent primary molar and no infraocclusion in the others, failure to determine the occlusal plane, significant loss of morphology in the primary molars, an orthodontic appliance in the area or an unclear DPT. Repeatability was estimated by measuring 146 DPTs twice.

#### Transposition

Transpositions of a canine and first premolar in the maxilla and a canine and lateral incisor in the mandible were included. Teeth were classified as transposed if their crowns had crossed each other as seen in the DPT despite the presence of adequate space in the dental arch. This was assessed by KK and confirmed by JR.

#### Distal angulation of unerupted mandibular second premolars

A mandibular second premolar was classified by KK as distally displaced if the long axis of the developing premolar intersected with the mesial border of the mandibular first molar (adapted from Baccetti et al. [20]). The exclusion criteria, confirmed by JR, were an emerged mandibular second premolar, mandibular second premolar root development that had not yet started, or the absence of the mandibular first molar.

### Features of maxillary canines

#### Overlapping and inclination

Overlapping of a maxillary canine crown with the lateral incisor root (overlapping) and inclination (α) (inclination) were measured on the DPTs by KK using the neaView Radiology software (Neagen Oy) (see Figure 1 in Ristaniemi et al. [7], modified from Ericson and Kurol [10]). Overlapping was classified as Grade 0 (no overlapping), Grade 1 (crown of the canine covering half or less of the width of the lateral incisor root) and Grade 2 (crown of the canine covering more than half of the width of the lateral incisor root). Overlapping was not determined in dentition involving an absent lateral incisor. The inclination was measured as the angle between the mid-sagittal suture of the maxilla and the mid-axis of the canine, where the latter was defined by reference to the pulp chamber. In cases of crooked roots and rotated teeth, the average tooth axis was adjusted by reference to the crown and root. The exclusion criteria were an emerged canine, complete primary or permanent dentition, orthodontic treatment at the time of the DPT or an unclear DPT. The measurements were assessed for repeatability by measuring 31 DPTs twice.

**Figure 1:**
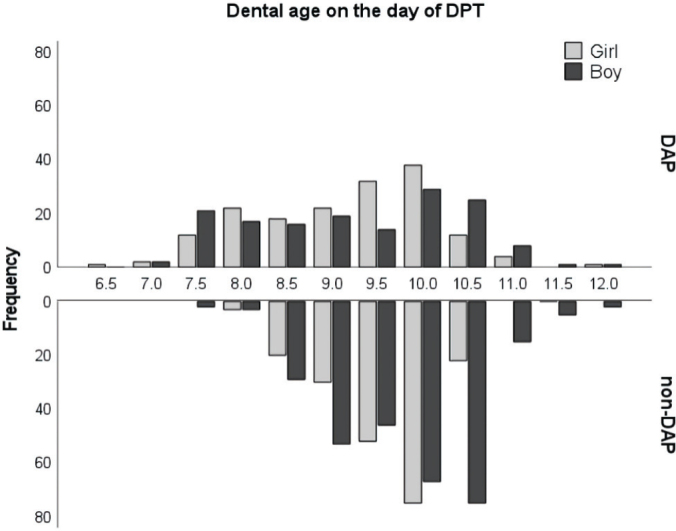
Distribution of DAP children (*n* = 392) and non-DAP children (*n* = 499) by dental age in years. DAP: Dental Anomaly Patterns.

#### Developmental stages of the maxillary canines and lateral incisors

The root development stages of the maxillary canines were assessed from the DPTs by WR following the method of Nolla [36] and scaled as Stage 1 (root formation started), Stage 2 (one-third of the root length completed), Stage 3 (half of the root length completed), Stage 4 (at least two-thirds of the root length completed) and Stage 5 (root completed, apex open/closed). The exclusion criteria were oligodontia (> six missing teeth) or an unclear DPT. Repeatability was estimated from 65 randomly selected DPTs (130 maxillary canines).

The development stages of the lateral incisors in the maxilla were assessed by WR from the DPTs and categorized as incomplete or complete (Stage 5). The exclusion criteria were a peg-shaped lateral incisor, an absent lateral incisor, oligodontia (> six missing teeth) or an unclear DPT.

#### Treatment

Orthodontic treatment necessitated by the eruption of a maxillary canine was carried out according to a treatment plan made by a senior orthodontist and information on this treatment was collected retrospectively by JR, KK and RL from the dental records found in the health centre’s paper archives or software after DPT and until the maxillary canine had been caused to erupt. The exclusion criteria for this variable were an emerged canine (based on DPT and/or dental records), oligodontia (> six absent teeth), poor quality of the DPT, odontoma or a cyst in the maxillary canine area or transposition of a maxillary canine and the first premolar. Orthodontic treatment carried out before DPT or after the eruption of a maxillary canine was not included. The forms of treatment were categorised as shown in Table 1.

**Table 1:** Maxillary canines categorized according to treatment.

Treatment need	Definition
**Natural eruption of a canine**	
No treatment	
No treatment	No treatment for maxillary canine
Follow up	No treatment for maxillary canine, follow-up carried out
**Treated maxillary canines**	
Early treatment	
Interceptive treatment	Extraction of a primary canine and/or interceptive slicing of a primary second molar and at the same time extraction of a primary first molar if needed due to eruption of a maxillary canine
Early headgear	Dental headgear applied with an Interlandi set-up during mixed dentition before or during eruption of the maxillary canines, possibly including other interceptive procedures
Late treatment	
Orthodontic treatment	Mainly surgical exposure of a maxillary canine and traction with a buccal bow (‘TMA 0.016 × 0.018’) from the transpalatal arch and later alignment with a fixed appliance. Sometimes surgical exposure alone or only traction with a fixed appliance was enough.
Treatment for crowding	Extraction of a permanent first premolar and/or treatment with a fixed appliance for relieving severe crowding especially for a maxillary canine. In some cases the extracted tooth may be a permanent second premolar.

### Inclusion criteria

The inclusion criteria for this study were chronological age 8.5–10.5 years at the time of the DPT, dentition in the mixed stage and no syndromes of clefts (study material). The group of children with DAP (DAP children) included those with at least one dental developmental abnormality of the kind studied here as being involved in DAP [17] as seen in the DPT, while the group for comparison (non-DAP children) was selected from the rest of the children, who had no such dental developmental abnormalities in their DPT.

### Statistics

The distributions of the variables studied here were described in terms of frequencies and percentages, and comparisons of the variables between the genders were performed using Pearson’s Chi-square test. Comparisons between the maxillary canines of the DAP and non-DAP children and between the treatment subgroups were performed with either Pearson’s Chi-square test or Fisher’s exact test. The normality of continuous variables such as angles and chronological and dental ages was assessed visually using histograms. Mean chronological and dental ages were analyzed with the independent samples *t*-test.

The single associations of independent DAP features and gender with the response variables (no treatment, early treatment, late treatment, all treatments) were determined with crude logistic regression models, while adjusted logistic regression models were used to check the associations of all DAP features and gender with the response variables in the study material. Statistically significant two-way interaction terms were checked during the formation of the models, the strength of each association being illustrated with an odds ratio and 95% confidence interval. The logistic regression models were resolved using the SAS glimmix procedure with random effect, to take account of children having two canines in the data.

The statistical analyses were performed using IBM SPSS Statistics (version 28.0) and SAS Enterprise guide 7.1. *P*-values <0.05 were considered statistically significant. Repeatability was assessed using Cohen’s kappa or intra-class correlation (ICC).

## Results

### Repeatability

Intra-rater repeatability was assessed for infraocclusion (*k* = 0.837), dental age (examiner 1 ICC = 0.789 and examiner 2 ICC = 0.945), overlapping (d.13 *k* = 0.917, d.23 *k* = 0.849), inclination (d.13 ICC = 0.933, d.23 ICC = 0.922) and MxC root development (Stages 1–5) (*k* = 0.777), while inter-rater reliability was assessed for dental age (ICC = 0.871). The assessments proved to be reliable in terms of their repeatability.

### DAP and non-DAP children

The study material consisted of 1,315 DPTs representing 619 girls and 696 boys aged 8.5–10.5 years. The inclusion criteria for DAP children were met by 392 children (207 girls and 185 boys), their mean chronological age at the time of the DPTs being 9.4 years (SD 0.4), with no gender difference (*p* = 0.980). The group of non-DAP children included 499 children (202 girls and 297 boys), with a mean chronological age of 9.3 years (SD 0.3) and no difference between the genders (*p* = 0.070). The mean chronological age did not differ between the DAP and non-DAP children (*p* = 0.881).

The dental ages of the DAP children varied from 6.5 to 11.9 years, with a mean of 9.1 years (SD 1.0) in the girls and from 7.1 to 11.9 years, with a mean of 9.2 years (SD 1.1) in the boys, whereas the dental ages of the non-DAP children varied from 7.9 to 10.7 years, with a mean of 9.6 years (SD 0.6) in the girls and from 7.7 to 12.0, with a mean of 9.8 years (SD 0.8) in the boys (Figure 1).

### Features of maxillary canines in the DAP and non-DAP children

The treatment provided could be assessed in the case of 1,269 canines, with no gender differences (*p* = 0.355). The maxillary canines of the DAP children had more often been subject to early treatment (*p* = 0.014) (Table 2), which was more often of an interceptive kind for the DAP girls (5.6% vs. 1.3%, *p* = 0.004), whereas the DAP boys more often had treatment with early headgear (5.9% vs. 2.5%, *p* = 0.022). Orthodontic treatment was provided more often for the non-DAP children (1.6% vs. 0.6%), but this difference was not statistically significant (*p* = 0.134).

**Table 2:** Distribution of maxillary canine treatment modes in DAP and non-DAP children by gender.

	Girls					Boys					All				*P* ^a^
	DAP		non-DAP		*P* ^a^	DAP		non-DAP		*P* ^a^	DAP		non-DAP		
	*n*	%	*n*	%		*n*	%	*n*	%		*n*	%	*n*	%	
**No treatment**	235	88.3	286	89.4		210	88.6	411	92.2		445	88.5	697	91.0	
**Early treatment**	22		16		0.127^b^	18		19		0.065^b^	40		35		0.014^b^
Interceptive treatment	15	5.6	4	1.3	0.004^b^	4	1.7	8	1.8	>0.999^c^	19	3.8	12	1.6	0.012^b^
Early headgear	7	2.6	12	3.8	0.477^b^	14	5.9	11	2.5	0.022^b^	21	4.2	23	3.0	0.243^b^
**Late treatment**	9		18		0.230^b^	9		16		0.821^b^	18		34		0.529^b^
Orthodontic treatment	2	0.8	7	2.2	0.198^c^	1	0.4	5	1.1	0.669^c^	3	0.6	12	1.6	0.134^b^
Treatment for crowding	7	2.6	11	3.4	0.602^b^	8	3.4	11	2.5	0.453^b^	15	3.0	22	2.9	0.847^b^

DAP: Dental Anomaly Patterns.

a Significances of differences between no treatment and the treatment subgroups;

b Pearson’s Chi-square test;

c Fisher’s exact test.

The distributions of maxillary canine features in the DAP and non-DAP children by gender are presented in Table 3. Within the total set of canines studied (*n* = 1,515), overlapping occurred more often in the girls (*p* = 0.026). The maxillary canines of the DAP children more often not only showed clear overlapping (Grade 2) but also no overlapping occurred more often (*p* = 0.006). Some (Grade 1) or clear overlapping occurred more often in the maxillary canines of the non-DAP boys than in the DAP boys (*p* = 0.023), whereas no difference in this respect was found between the girls (*p* = 0.078). The mean inclination angle was 13.4° (SD 7.5), but there was no difference in grouped inclination (*n* = 1,539) between the genders (*p* = 0.051) or between the DAP and non-DAP children (*p* = 0.724).

**Table 3:** Distribution of radiological features related to erupting maxillary canines in DAP and non-DAP children by gender.

	Girls				Boys				All			
	DAP		non-DAP		DAP		non-DAP		DAP		non-DAP	
	*n*	%	*n*	%	*n*	%	*n*	%	*n*	%	*n*	%
**Overlapping of canine** ^a^												
Grade 0	174	52.7	175	45.7	166	61.3	271	51.0	340	56.6	446	48.8
Grade 1	137	41.5	191	49.9	98	36.2	243	45.8	235	39.1	434	47.5
Grade 2	19	5.8	17	4.4	7	2.6	17	3.2	26	4.3	34	3.7
*P*-value^c^		0.078				0.023				0.006		
**Inclination of canine (** ^o^ **)**												
<15	198	58.9	229	59.5	168	60.2	347	64.4	336	59.5	576	62.3
15–19.9	73	21.7	81	21.0	63	22.6	107	19.9	136	22.1	188	20.3
20–24.9	34	10.1	44	11.4	35	12.5	56	10.4	69	11.2	100	10.8
≥25	31	9.2	31	8.1	13	4.7	29	5.4	44	7.2	60	6.5
*P*-value^c^		0.078				0.548				0.724		
**Canine root development** ^b^												
Stage 1	21	5.1	4	1.0	73	20.9	67	11.3	94	12.3	71	7.1
Stage 2	224	54.4	223	57.7	225	64.3	423	71.5	449	58.9	656	65.9
Stage 3	112	27.2	140	34.7	40	11.4	85	14.4	152	19.9	225	22.6
Stage 4	52	12.6	27	6.7	12	3.4	17	2.9	64	8.4	44	4.4
Stage 5	3	0.7	0	0.0	0	0.0	0	0.0	3	0.4	0	0.0
*P*-value^c^		<0.001				<0.001				<0.001		
**Lateral incisor development** ^b^												
Incomplete	324	80.6	314	79.7	320	92.0	514	87.7	644	85.9	828	84.5
Complete	78	19.4	80	20.3	28	8.0	72	12.3	106	14.1	152	15.5
*P*-value^c^		0.750				0.043				0.426		

DAP: Dental Anomaly Patterns.

a Grade 0 (no overlapping), Grade 1 (≤ 1/2 overlapping) and Grade 2 (> 1/2 overlapping);

b Division is based on developmental stages as defined by Nolla’s method [36];

c Pearson’s Chi-square test.

The stage of canine root development yielded Nolla’s values [36] in the range 6.0–9.0 (*n* = 1,758) and was significantly more advanced in the girls (*p* < 0.001), as was also the case with the stage of lateral incisor development (*n* = 1,730) (*p* < 0.001). The root development stage of the canines varied among the DAP and non-DAP children, with the DAP children’s canines more often in either an early (Stage 1) or later stage (Stages 4–5), whereas those of the non-DAP children were mainly at Stages 2 and 3 (*p* < 0.001). The stage of lateral incisor development was more often incomplete in the DAP boys (*p* = 0.043) but did not differ among the girls (*p* = 0.750).

### Associations between DAP features and maxillary canine treatment

In crude logistic regression analysis of the study material, the absence of teeth (OR 2.09, 95% CI 1.01–4.35) and delayed dental age were associated with the provision of treatment (OR 2.39, 95% CI 1.04–5.49), within which delayed dental age was especially associated with early treatment (OR 3.29, 95% CI 1.08–9.99) (Table 4). The adjusted logistic regression models for the study material did not give any associations between the DAP features studied here and the treatment groups (Table 4).

**Table 4:** Crude logistic regression analysis and logistic regression models for associations between DAP features^a^ and maxillary canine treatment groups in the study material.

	Early treatment				Late treatment				All treatments			
	Crude		Model		Crude		Model		Crude		Model	
	OR	95% CI	OR	95% CI	OR	95% CI	OR	95% CI	OR	95% CI	OR	95% CI
Gender boy (ref. Girl)	0.83	0.47–1.47	0.89	0.44–1.81	1.03	0.59–1.80	0.72	0.33–1.56	0.93	0.62–1.41	0.81	0.46–1.41
Absent teeth (ref. no)	2.57	0.98–6.80	3.49	0.93–13.09	1.52	0.53–4.32	2.25	0.40–12.56	**2.09**	**1.01–4.35**	2.93	0.94–9.12
Peg-shaped MxI2 (ref. no)^b^	4.03	0.68–23.75	2.27	0.29–17.54	0.83	0.07–10.28	–	–	2.50	0.61–10.20	1.41	0.21–9.53
Delayed dental age (ref. normal)	**3.29**	**1.08–9.99**	2.53	0.83–7.69	1.53	0.46–5.10	0.74	0.14–3.92	**2.39**	**1.04–5.49**	1.89	0.72–4.95
Infraocclusion (ref. no)	1.15	0.53–2.50	1.39	0.58–3.31	0.36	0.11–1.14	0.35	0.10–1.27	0.78	0.41–1.48	0.87	0.41–1.84
Distal angulation of MnP2 (ref. no)	1.09	0.34–3.55	0.62	0.10–3.94	1.04	0.31–3.52	2.10	0.51–8.74	1.07	0.45–2.55	1.21	0.38–3.82
DAP feature (ref. no)^c^	1.81	0.96–3.42			0.84	0.40–1.76			1.32	0.81–2.16		

DAP: Dental Anomaly Patterns.

a Prevalences of DAP features in the study material are presented in Table 2 [22];

b Peg-shaped MxI2 was excluded from the logistic regression model for late treatment;

c Transposition was excluded from the analysis and models

Results are illustrated for the maxillary canines, so that the DAP feature was the same for both maxillary canines. Statistically significant values (*p* < 0.05) are in bold.

## Discussion

The present report on radiological features of the maxillary permanent canines and the treatment carried out in relation to the occurrence of certain dental developmental abnormalities [17, 22] is based on the same representative age cohort of DPTs as the researchers’ earlier studies of maxillary canine eruption [7, 9].

The overlapping of maxillary canines was found to vary significantly more in the DAP children, and these more often had either clear overlapping or no overlapping at all. The non-DAP boys had either clear overlapping or some overlapping significantly more often than did the DAP boys. Thus, we can only partly accept our first hypothesis with regard to more pronounced overlapping in the canines of children with the dental developmental abnormalities studied here. On the other hand, there was no significant difference between the DAP and non-DAP children in the inclination angle of the maxillary canines, so that this part of the hypothesis must be rejected.

Our second hypothesis, concerning the treatment of maxillary canines was partly confirmed, since the canines of the children with dental developmental abnormalities received early treatment significantly more often. Interceptive treatment was carried out significantly more often in the DAP girls, while the DAP boys tended to receive early headgear treatment. Late treatment was less related to the occurrence of DAP features, and therefore our hypothesis concerning this must be rejected. The number of children in orthodontic treatment, including surgical exposure, was small, but orthodontic treatment was mentioned over twice as often (1.6% vs. 0.6%) in the children without dental developmental abnormalities.

The children in the present series were equally divided in terms of chronological age both between the genders and between the DAP and non-DAP groups. Delayed dental age is one feature involved in DAP [17], and it has also been connected with eruption disturbances in the maxillary canines [21, 37, 38]. Delayed dental age was found in this material to be associated with early treatment, suggesting that early treatment should be started early enough, i.e. after the first mixed stage. As expected, the dental ages of the children with DAP were later and varied by as much as five and a half years in these children as opposed to four and a half years in the non-DAP children. These findings reflect differences in occlusal development and underline the importance of evaluating the mixed dentition stage as part of the overall dental examination.

It has been found earlier that canine root development neither differ between normal and ectopic canines [6] nor between naturally erupting and treated canines [9]. In this study the early stage of canine root development in the DAP children can be attributed to the delayed dental age, our new finding was that advanced root development (at least two-thirds completed) occurred more often in the DAP children than in the non-DAP children.

A couple of years before the maxillary canines erupt into the oral cavity the development of the lateral incisors is in most cases incomplete and more often complete in girls [7, 9]. Thus, a comparison of the DAP and non-DAP groups showed lateral incisor development to differ only in the boys, whereas those with DAP more often had incomplete lateral incisors. The background to this may be that delayed dental age is one of the studied DAP features.

As a feature involved in DAP, absent teeth were slightly associated with the treatment of a maxillary canine. This is in line with earlier findings that children with absent teeth more often presented with ectopic eruption of a maxillary canine [16, 19].

One limitation of this work can be seen in its retrospective design and the cross-sectional sample represented by the DPTs. Due to variation in dental development, some of the DAP features might have been visible before or after the DPTs had been taken. In the light of the senior orthodontist’s treatment plans, some children had received orthodontic treatment for the primary dentition or during the early mixed stage (before the DPT), probably in the form of a Quad Helix for posterior cross-bite, braces on the upper incisors to close a large medial diastema, slicing of the primary canines mesially in the case of minimum space deficiency during the eruption of the lateral incisors, or elastics for a first molar cross-bite. The method used here for dental age estimation [35] is gender-specific, and thus it should be observed that the dental age differences between the genders do not directly reflect gender differences in dental development stages. The subsamples used for assessing the treatment provided for the maxillary canines were substantially small, another limitation that must be considered when reflecting on the results.

Our findings with regard to the treatment provided represent a successful follow up and early treatment of maxillary canines in the children with dental developmental abnormalities. Nevertheless, the results underline the importance of monitoring the erupting canines in children with and without dental developmental abnormalities. The oral examinations for the present children were mainly carried out at one primary school level, and it is noticeable that dental age varied markedly. This may suggest that early treatment was completed in time, as dental development was later in the DAP children. It is necessary to monitor the erupting maxillary canines after the first mixed stage in the dentition, and by evaluating the child’s dental developmental stage it is possible to enable early treatment. Early treatment after the first mixed stage, e.g. with headgear [14, 15], can ensure proper space conditions for the canines to erupt. Similar studies will be needed to verify these results and support clinical decision-making.

## Conclusion

A couple of years before the eruption of a maxillary canine the DPT showed that:

- Either no overlapping or clear overlapping occurred more often in the DAP children, while canine inclination did not differ between the DAP and non-DAP children.
- The root development stage of the canines was more often either at the beginning or advanced in the DAP children.

The canines of the DAP children had more often undergone early treatment and delayed dental age as a DAP feature was associated with this early treatment. This enabled the proper timing of early treatment in relation to the dental developmental stage.
